# βArrestin2 Mediates Renal Cell Carcinoma Tumor Growth

**DOI:** 10.1038/s41598-018-23212-w

**Published:** 2018-03-20

**Authors:** Jude Masannat, Hamsa Thayele Purayil, Yushan Zhang, Michelle Russin, Iqbal Mahmud, Wanju Kim, Daiqing Liao, Yehia Daaka

**Affiliations:** 1 Department of Anatomy and Cell Biology, University of Florida College of Medicine, Gainesville, FL 32610 USA; 2 Present Address: Moffitt Cancer Center, Tampa, FL USA; 3 Present Address: Stephenson Cancer Center, University of Oklahoma, Oklahoma City, OK USA

## Abstract

Renal Cell Carcinoma (RCC) is one of the most lethal urological cancers worldwide. The disease does not present early clinical symptoms and is commonly diagnosed at an advanced stage. Limited molecular drivers have been identified for RCC, resulting in the lack of effective treatment for patients with progressive disease. Ubiquitous βArrestin2 (βArr2) is well established for its function in the desensitization and trafficking of G protein-coupled receptors. More recently, βArr2 has been implicated in the regulation of fundamental cellular functions, including proliferation and invasion. We used bioinformatic and genetic approaches to determine role of βArr2 in RCC tumor growth. Analysis of published human datasets shows that ARRB2 (gene encoding βArr2) expression is increased in RCC tumor compared to normal tissue and that high levels of ARRB2 correlate with worse patient survival. Experimentally, we show that knockout of ARRB2 decreases rate of RCC cell proliferation and migration *in vitro* and xenograft tumor growth in animals. Mechanistically, βArr2 regulates c-Src activity, Cyclin A expression and cell cycle progression that are involved in tumor growth. These results show that βArr2 is a critical regulator of RCC tumor growth and suggest its utility as a potential marker and drug target to treat advanced disease.

## Introduction

Kidney cancer is one of the top-ten leading cancers in the US with few effective treatments and high lethal consequences. Kidney cancer incidence and mortality rates are on the rise. In the US, a decade ago 31,900 cases of kidney cancer were diagnosed and 11,900 patients died from the disease^1^. This year, the estimated number of new cases has doubled to 63,990 with 14,400 estimated deaths^2^. Worldwide, RCC is diagnosed in about 300,000 people, and causes more than 100,000 deaths annually^3,4^. Hence, identification of molecular culprits responsible for disease initiation and progression is urgently needed to address the ever-growing number of kidney cancer cases.

The majority (80–90%) of kidney cancers are classified histologically as renal cell carcinoma (RCC) that can be subdivided into clear cell (ccRCC) and non-clear cell (nccRCC) RCC subtypes^5^. The standard of care for patients diagnosed with organ-confined RCC is surgical resection of the tumor mass or whole kidney. However, this treatment may not be an option for patients with poor overall health or advanced disease, which decreases the overall 5-year life expectancy to around 10%^6^. Also, about one third of RCC cases are diagnosed at the metastatic stage where mortality rates are the highest among any adult urological cancer^7,8^. Indeed, RCC exhibits a spectrum of genetic mutations and often the available therapies, which target receptor tyrosine kinases such as vascular endothelial growth factor receptor and intracellular signaling hubs like the mammalian target of rapamycin, fail within a year of treatment^9^.

There are two βArrestin proteins, namely βArrestin1 (βArr1) and βArrestin2 (βArr2), that are ubiquitous and exhibit a high degree of sequence homology and functional redundancy (reviewed in refs^10,11^). In addition to their well-established roles in G protein-coupled receptor desensitization and internalization, βArrestins have been reported to scaffold signal transduction mediators involved in fundamental cellular functions, including growth and migration^10,12–16^. For example, βArr1 is overexpressed in gastric cardiac adenocarcinomas^17^, promotes prostate cancer by modulating androgen receptor activity^16^, interacts with the tyrosine kinase c-Src in colorectal cancer^15^, and induces rapid xenograft tumor progression in mouse models^18^. Likewise, βArr2 mediates the initiation and progression of myeloid leukemia through the activation of Wnt signaling^19^, forms complex with c-Src that promotes epidermal growth factor receptor (EGFR) transactivation^20^, and induces tumor cell proliferation and metastasis^21^. It has been reported that invasive breast cancer cell lines express high levels of βArr2, which was suggested to regulate the cancer cell proliferation and invasion^22^. However, it was also reported that the downregulation of βArr2 promotes hepatocellular carcinoma tumor invasion^23^. These seemingly contradictory results imply that βArr2 function may be cell context- and cancer type-dependent.

While βArr1 and βArr2 exhibit high degree of sequence homology and function overlap, their subcellular distribution is distinct. βArr1 is expressed in the cytosol and nucleus and has been shown to exert its mitogenic function, at least in part, through the regulation of gene expression^10,11,16,24^. Less is known about how βArr2, which is strictly detected in the cytosol, regulates mitogenesis. Moreover, to date no studies have been reported on the role of βArr2 in RCC. Here, we show that βArr2 controls c-Src activation and Cyclin A expression and regulates RCC localized and metastatic tumor growth.

## Results

### ARRB2 is abundantly expressed in human RCC

**Figure 1:**
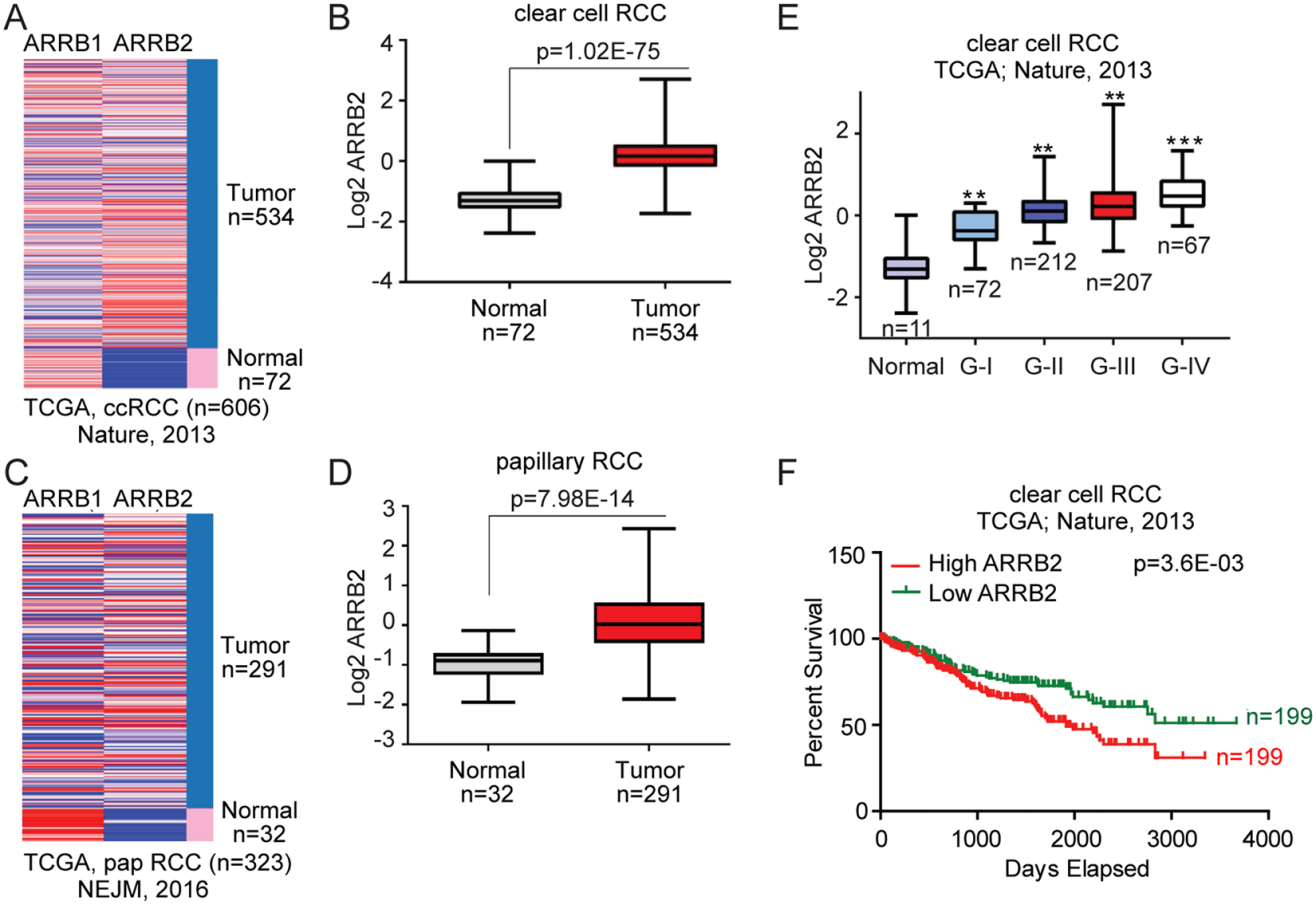
ARRB2 is abundantly expressed in human RCC. (**A**) Heat map generated from The Cancer Genome Atlas (TCGA) database showing relative expression of ARRB1 and ARRB2 in normal and ccRCC patients. (**B**) Box and whisker plots for ARRB2 gene expression in ccRCC patient compared to normal. Log2-normalized read count [RNA-seq by expectation-maximization (RSEM)] is shown. (**C**) Heat map showing ARRB1 and ARRB2 genes expression in normal and papillary carcinoma patients from TCGA. (**D**) Box and whisker plots for ARRB2 in papillary RCC patient compared to normal. (**E**) Box and whisker plots represent ARRB2 gene expression for tumor samples stratified according to histologic grade. (**F**) Kaplan–Meier survival plot represents overall survival of ccRCC patients in TCGA for RCC patients categorized according to ARRB2 gene expression (high vs low in comparison to the median expression). For panels (**B**,**D**) and (**E**) the P values were calculated using a log-rank test. Box plot line (from top to bottom): maximum; Q3, third quartile; median; Q1, first quartile; and minimum. P values were calculated by using two-tailed paired Student’s t test. Data shown are mean ± SEM, **P < 0.001 and ***P < 0.0001.

To determine potential significance of βArr2 in RCC, we first analyzed available human datasets of ccRCC patients for ARRB1 and ARRB2 genes expression. We found that ARRB2, but not ARRB1, gene is significantly more expressed in ccRCC tumor compared to normal tissue (Fig. 1A,B). Similar results were observed for other kidney cancer subtypes, including papillary (Fig. 1C,D) and chromophobe (Supplemental Fig. S1) RCC that were reported in additional datasets. Notably, ARRB2 levels correlated with ccRCC disease stage (Fig. 1E), and survival data showed that patients (diagnosed with ccRCC) with high ARRB2 expression levels had significantly lower survival outcomes compared to patients with low ARRB2 expression (Fig. 1F). Collectively, these results demonstrate that increased ARRB2, but not ARRB1, expression correlates with advanced ccRCC stage and decreased patient survival.

### βArr2 expression is upregulated in aggressive human RCC cell lines

**Figure 2:**
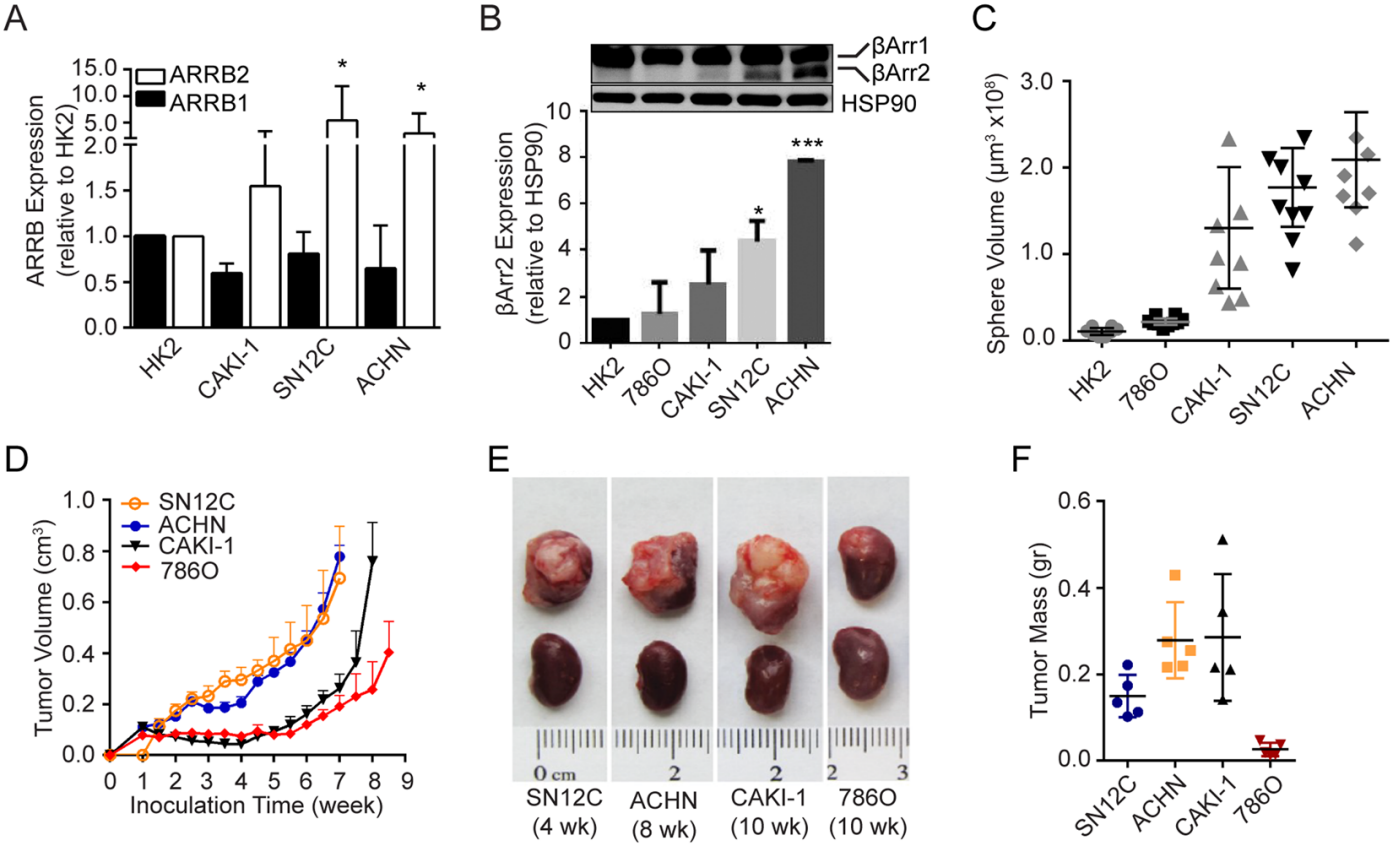
βArr2 expression is upregulated in metastatic human RCC cell lines. (**A**) Relative expression of ARRB1 and ARRB2 in RCC cells lines. Gene expression levels in CAKI-1, SN12C, ACHN and HK2 cells were measured by real-time PCR and are presented as fold change relative to HK2 cells. *P < 0.05. (**B**) Relative expressions of βArr proteins in RCC cells. Protein levels of βArr1 and βArr2 in HK2, 786O, CAKI-1, SN12C, and ACHN cells were analyzed by Western blot (top) and βArr2 levels were quantified by image J (bottom) and are presented as fold change relative to HK2 cells (that were arbitrarily assigned a value of 1). *P < 0.05, ***P < 0.0001. (**C**) Sphere growth assay. RCC cells (200) were seeded in matrigel and allowed to grow for 22 days. Sphere sizes were captured and average volume of the largest 10 spheres for each cell line were plotted. (**D**) Subcutaneous tumor growth. Cells (1 × 10^7^) were inoculated in the flank of athymic nude mice. Tumor growth was monitored by measuring the length and width of the tumors, and tumor volume was calculated using the formula: V = 0.524 × length × width^2^ (n = 6 animals per cell line). (**E**) Subrenal tumor growth. Cells (1 × 10^6^) were embedded in collagen pellet and implanted under the renal capsule of athymic nude mice. Tumors were harvested at 4 to 10 weeks after implantation. Images represent n = 5 mice. (**F**) Subrenal tumor weight from panel E were calculated by subtracting weight of the contralateral normal kidney from the weight of kidney bearing tumor. Please note the variable implantation times. Tumor growth experiments were repeated twice with similar results.

To test the functional relevance of increased ARRB2 in advanced RCC, we analyzed ARRB2 (and ARRB1 as a control) expression in commonly used RCC cell lines, including CAKI-1, 786O, SN12C, and ACHN. The human kidney epithelial cell line HK2 (immortalized proximal tubule epithelial cells from normal adult kidney) served as a control^25^. The results show that ARRB2, but not ARRB1, expression was significantly higher in SN12C and ACHN, in comparison to HK2 cells (Fig. 2A). Concordantly, whereas βArr1 protein levels were similar amongst the cell lines, βArr2 protein levels were highest in SN12C and ACHN cells, exhibiting 5–8 fold increases above HK2 cells (Fig. 2B).

Next, we characterized these cell lines for their growth properties using *in vitro* and *in vivo* assays. Results showed that SN12C and ACHN cells formed faster growing spheres in matrigel (Fig. 2C) and tumors in animals (Fig. 2D–F), in comparison to HK2, 786O and CAKI-1 cells. These results lead us to conclude that increased βArr2 levels correlate with high proliferative capacity of RCC cells.

### Knockout of βArr2 inhibits the cell proliferation

**Figure 3:**
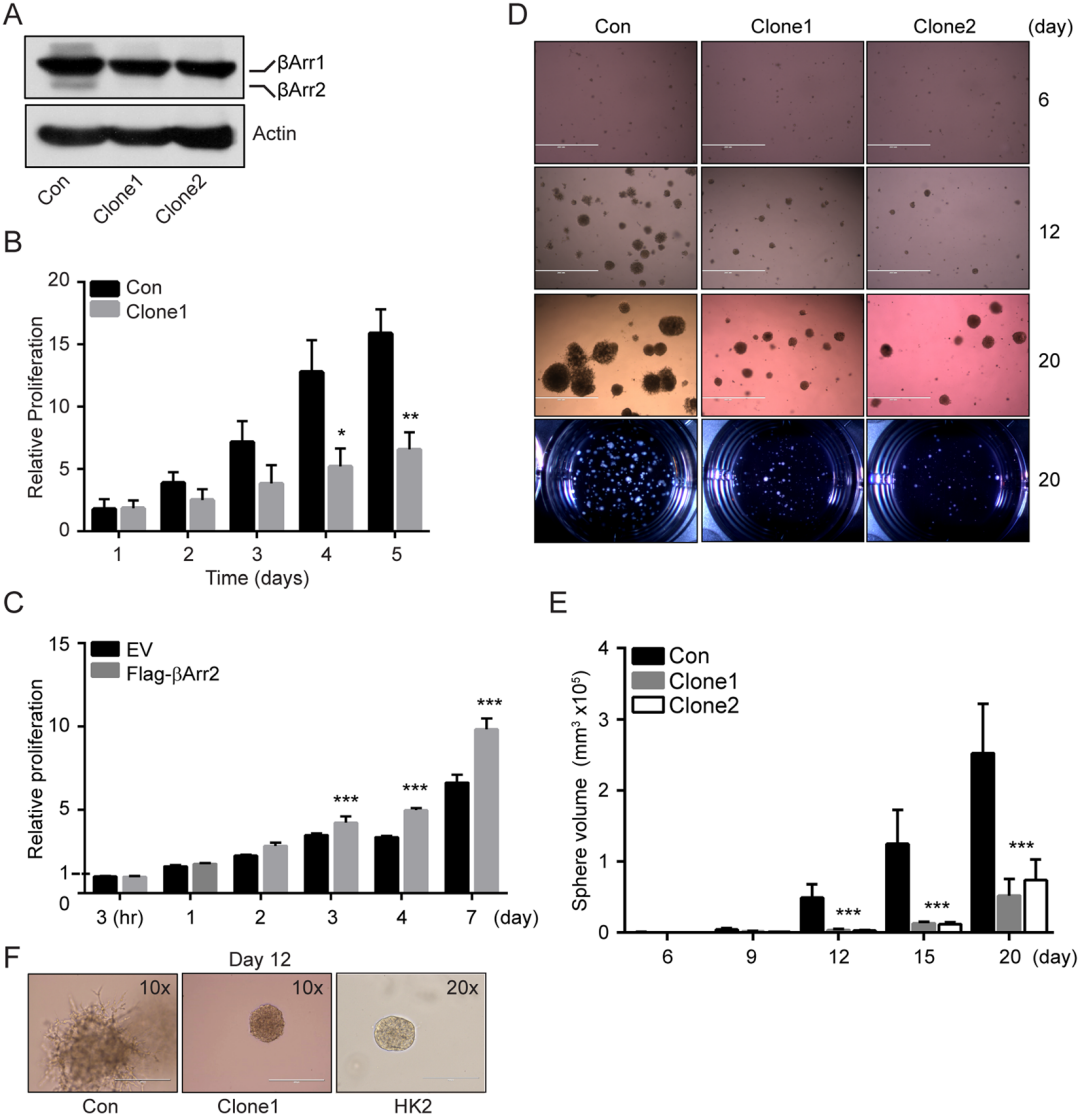
Knockout of βArr2 inhibits cell proliferation. (**A**) βArr2 knockout in SN12C cells. SN12C cells were infected with lentivirus CRISPR/Cas9 containing sgRNA targeting exon 3 (Arrb2ex3-14; Clone1) or exon 4 (Arrb2ex4-12.19; Clone2) respectively. The expression levels of βArr2 in knockout cells were analyzed by Western blot. Control (Con) SN12C cells were infected with lentivirus CRISPR/Cas9 that contains no sgRNAs. (**B**) Cell proliferation assay. Bars represent the average proliferation rate of control and βArr2^ko^ Clone1 cells over time relative to the number of control cells after 1 day of incubation. (**C**) Cell proliferation assay. Bars represent the fold change in proliferative rate of empty vector (EV) and Flag-βArr2 CAKI-1 cells relative to the number of EV CAKI-1 cells after 3 hr incubation. (**D**) Sphere growth assay. Control or βArr2^ko^ Clone1 and Clone2 single (500) cells were seeded in matrigel and allowed to grow for 20 days. Sphere sizes were captured every 3 days till day 15 and at endpoint day 20. Images shown are representative of growth at days 6, 12, and 20. Bird’s eye view of the wells at the end point are also shown. (**E**) Bars represent the average volume of 20 spheres from panel D analyzed at the indicated time point. (**F**) Images of spheres (control and βArr2^ko^ Clone1) at 10× magnification and HK2 sphere at 20× magnification on day 12. For panels B, C and E, data shown represent the average ± SD for three independent trials. *P < 0.05, **P < 0.001 and ***P < 0.0001.

We created stable βArr2 knockout SN12C lines using CRISPR/Cas9 system. Cells were infected with a single lentiviral CRISPR/Cas9 plasmid containing specific sgRNAs that targeted exon 3 or exon 4 of ARRB2 gene. Control cells were also generated using the same CRISPR/Cas9 plasmid that, however, lacked sgRNA. Single cell expansion yielded multiple clones, including ARRB2ex3–14 (βArr2^ko^ Clone1) and ARRB2ex4–12.19 (βArr2^ko^ Clone2) that did not show detectable βArr2 protein on Western blot, but exhibited normal expression of endogenous βArr1 protein (Fig. 3A).

To elucidate role of βArr2 in the RCC cell growth, we used the control and βArr2^ko^ clones in *in vitro* cell proliferation assays. Results show that the knockout of βArr2 expression decreased the proliferation rate of SN12C cells cultured in serum-containing growth medium (Fig. 3B). To add support to this observation, we engineered CAKI-1 cells (that have low levels of endogenous βArr2; Fig. 2B) to express empty vector (EV) and Flag-tagged βArr2 (Flag-βArr2) (Supplemental Fig. S2A). We observed that the forced expression of βArr2 significantly increased proliferation rate of the Flag-βArr2 CAKI-1 cells, in comparison to the control EV cells (Fig. 3C).

We used sphere formation in matrigel assay as another way to further implicate βArr2 in RCC growth. Results show that whereas control SN12C cells formed large numbers of fast-growing spheres, cells with βArr2^ko^ formed fewer and slow-growing spheres (Fig. 3D,E). In addition to differences in the sphere growth rate (Fig. 3D,E), we also observed morphological differences between the control and βArr2^ko^ colonies growing in matrigel (Fig. 3F). We observed that outer cells in control colonies displayed spike-like extensions whereas colonies originating from cells with βArr2^ko^ had smooth edges, like those of the non-tumorigenic HK2 cells (Fig. 3F).

### βArr2 mediates RCC cell migration and invasion

**Figure 4:**
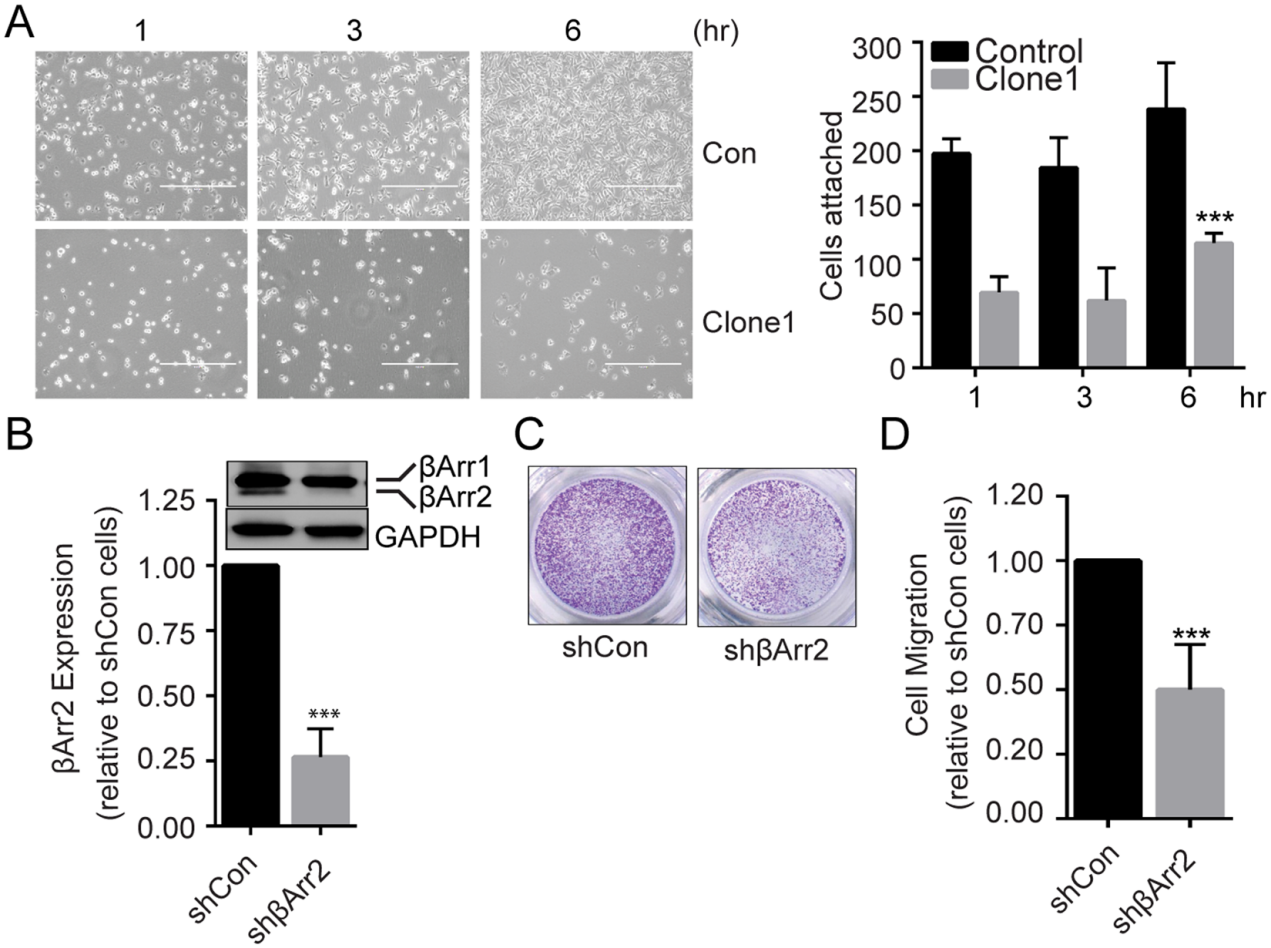
βArr2 mediates RCC cell migration and invasion. (**A**) Cell attachment assay. Control and βArr2^ko^ Clone1 (2.5 × 10^5^) cells were seeded on fibronectin-coated plates, and cell attachment was observed under a phase contrast microscope at 1, 3, and 6 hr after seeding. White halos indicate semi-attached or floating cells. Bar plot showing attached cell numbers counted (10×) at each time point from three independent trials (right panel). (**B**) Knockdown of βArr2 in RCC cells. SN12C cells were infected with lentiviral vector containing shβArr2 or empty pLKO plasmid. Cell lysates were analyzed for βArr2 expression by Western blot (top), and band intensities were quantified by image J (bottom). (**C**) Cell migration assay. Control and βArr2 knockdown cells (3 × 10^4^) were starved overnight in 0.1% BSA and seeded onto transwell migration inserts. Cells that migrated through the transwell inserts over the 1% FBS gradient after 24 hr were stained with crystal violet and imaged. (**D**) Migrated cells in randomly-selected five fields were counted and plotted relative to control cells. Images shown are representative of three independent trials. For panels A, B and D, ***P < 0.0001.

The effect of βArr2 knockout on sphere morphology suggests a role in cell invasion and migration. Cell attachment to surrounding matrix is a prerequisite for the cell migration and invasion, and we used *in vitro* assay to test whether βArr2 plays a role in the cell attachment. Results show that as early as one hr after seeding, the number of attached βArr2^ko^ cells was significantly less than control cells (Fig. 4A). To overcome the possibility that the knockout of βArr2 yields compensatory mechanisms responsible for the reduction in cell attachment, we used shRNA in SN12C (Fig. 4B) and siRNA in ACHN (Supplemental Fig. S2B) cells to knockdown βArr2. We measured rate of cell migration in transwell chambers and observed a 50% reduction in the migration of both cell types when βArr2 was knocked down, in comparison to respective controls (Fig. 4C,D and Supplemental Fig. S2C,D). We also tested impact of βArr2 on the cell invasion of matrigel and could show that the knockdown of βArr2 inhibited ACHN cell invasion by 60% compared to control cells (Supplemental Fig. S2E). Together, these results establish a role for βArr2 in RCC cell migration and invasion.

### RCC localized and metastatic tumor growth is controlled by βArr2

**Figure 5:**
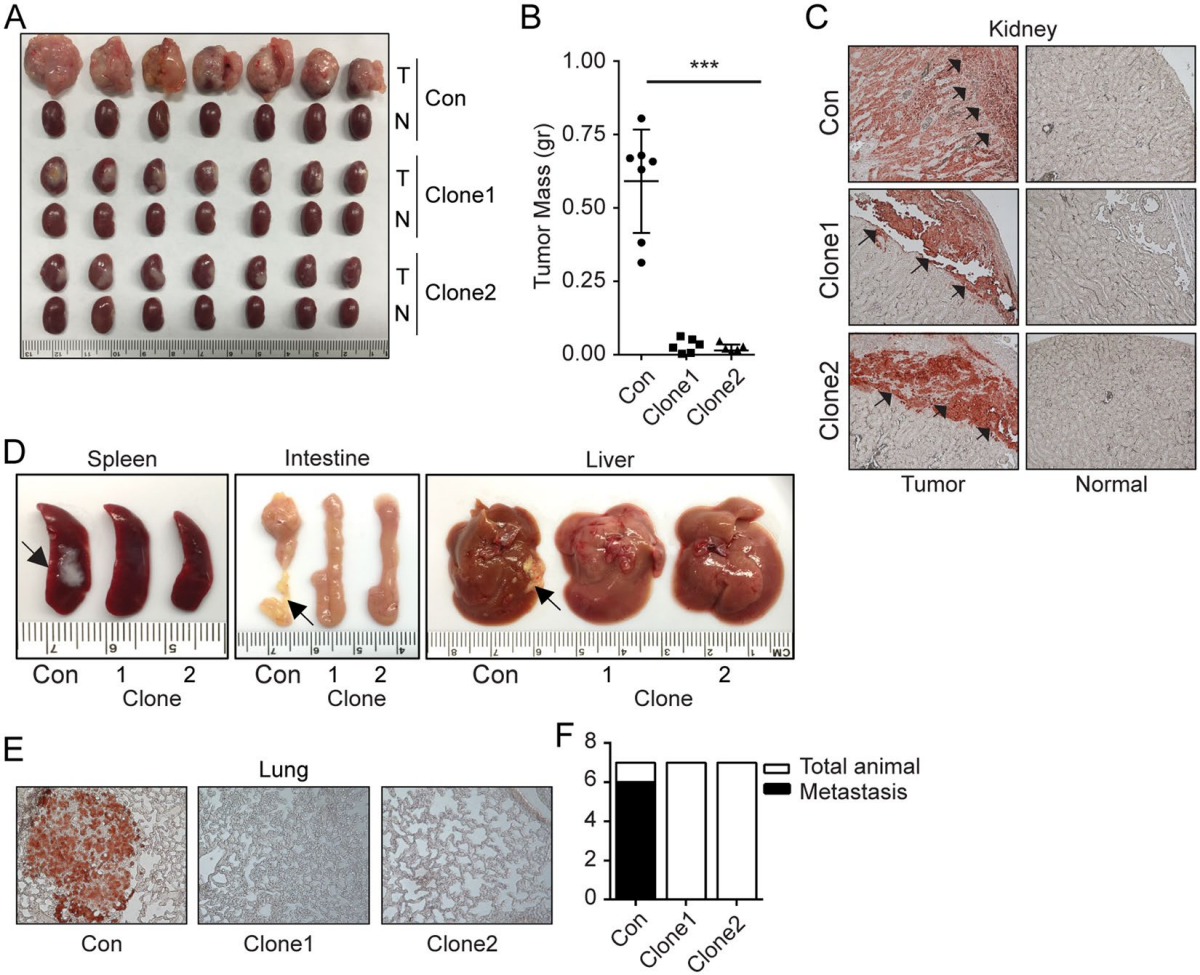
RCC tumor growth and metastasis are controlled by βArr2. (**A**) Subrenal tumor growth. Control and βArr2^ko^ Clone1 and Clone2 (1 × 10^6^) cells were embedded in collagen and implanted under the renal capsule of athymic nude mice (n = 7 mice per cell line) and allowed to grow for 5 weeks. N, contralateral normal kidney and T, kidney harboring tumor. (**B**) Tumor weight was measured by subtracting the weight of N kidney from that of T kidney. ***P < 0.001. (**C**) Representative anti-human LDHA staining of kidney tissue sections showing the infiltration pattern of tumor cells into the mouse kidney. Arrows point to the edge of the cortex under the capsule. (**D**) Visible metastasis on spleen, liver, and intestine in control mice relative to mice implanted with βArr2^ko^ Clone1 and Clone2 SN12C tumors. (**E**) Representative images of anti-human LDHA staining of lung tissue. (**F**) Bar plot showing the number of total and metastasis-positive to the lungs.

Our initial studies established a positive correlation between RCC tumor growth rate and the expression levels of βArr2 (Fig. 2D–F). To directly implicate βArr2 in RCC tumor growth, we used subrenal capsule implantation approach to measure capacity of SN12C (control and βArr2^ko^; Fig. 5A) cells to form tumors in mice. Equal number of viable cells were implanted orthotopically in the subrenal capsule space of one kidney^26^. Tumors were allowed to grow for 5 weeks before animals were sacrificed and organs (tumor-implanted and contralateral normal kidney, lymph nodes, lung, spleen, liver, and intestine) harvested. For duration of the experiment, tumor size was monitored weekly by palpation^26^ and ultrasound imaging (Supplemental Fig. S3A). In agreement with the effect of βArr2 on cell (Fig. 3B,C) and sphere (Fig. 3D–F) growth, the results showed that tumors from βArr2^ko^ clones grew significantly less, in comparison to tumors originating from control cells (Fig. 5A,B).

Macroscopic analysis of the sacrificed animals evidenced presence of metastatic growth throughout the viscera of mice harboring control SN12C tumors, but not in animals implanted with βArr2^ko^ SN12C cells (Supplemental Fig. S3B). Concordantly, staining of kidney tissue sections with a human LDHA antibody revealed that tumors from βArr2^ko^ SN12C cells failed to invade through the cortex of the kidney, in contrast to control tumors where majority of the kidney cortex was infiltrated by the SN12C cells (Fig. 5C). Cancer cell local invasion and migration are a prerequisite for the tumor metastasis. Lymph node infiltration by cancer cells is an early mark for dissemination and metastatic growth. We observed an increase in renal lymph node size (Supplemental Fig. S3C,D) in animals implanted with control, but not βArr2^ko^ SN12C cells. The harvested organs were also inspected for the presence of visible metastatic nodules that were successfully seen in the spleen, intestine and liver obtained from animals implanted with control SN12C cells, but not with βArr2^ko^ SN12C cells (Fig. 5D). Moreover, the staining of lymph node (Supplemental Fig. S3E) and lung (Fig. 5E) tissues with human LDHA antibodies revealed presence of metastatic growth in control but not βArr2^ko^ tissues. For lungs, 6 out of 7 animals implanted with control SN12C cells showed metastatic growth, but no animals harboring βArr2^ko^ SN12C tumors evidenced the tumor growth at metastatic sites (Fig. 5F).

We repeated the subrenal capsule implantation experiment once more but this time extended duration of the experiment from the original 5 week to a 9-week period. The rationale was to test whether the prolonged implantation time would allow for the detection of localized and metastatic tumor growth. Unexpectedly, the almost doubling of the original experiment duration still failed to produce an increase in βArr2^ko^ tumor growth (Supplemental Fig. S4A,B). Furthermore, the animals did not show any visible metastatic tumor growth to the viscera or soft tissues, implying βArr2 is involved primarily in RCC tumor growth.

### βArr2 regulates cell cycle progression

**Figure 6:**
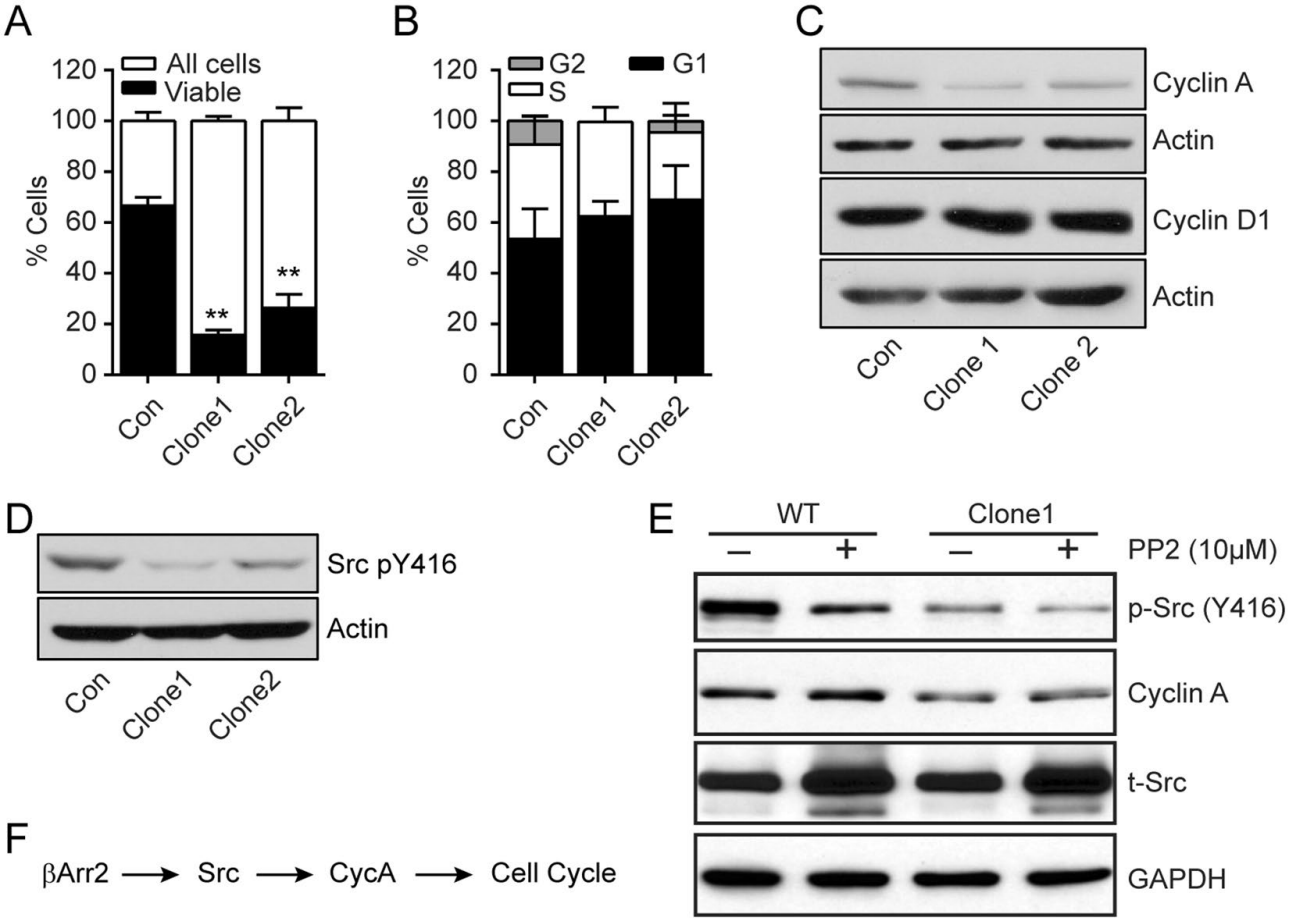
βArr2 regulates cell cycle progression. (**A**) Cell cycle analysis. Control and βArr2^ko^ (5 × 10^4^) cells were counted using LSRII flow cytometer and analyzed for cell cycle status. Bar-plot representation of the distribution of viable cells compared to total cells. **P < 0.001. (**B**) Bar-plot representation of the distribution of viable gated cells in G1 (P = 0.6311), S (P = 0.6974) and G2 (P = 0.0253) phase. (**C**) Representative images of Cyclin A and Cyclin D proteins expression in control and βArr2^ko^ cells. Actin served as a loading control. (**D**) Representative images depicting active c-Src. Cell lysates were blotted with anti-c-Src pY416 antibody. Actin served as a loading control. (**E**) Inhibition of c-Src depletes expression of Cyclin A. Cells were treated with PP2 (10 μM) for 24 hr followed by western blotting to detect the indicated proteins. Total c-Src (t-Src) and GAPDH served as loading controls. (**F**) Schema depicting cell cycle regulation.

βArr2^ko^ caused fundamental changes in RCC cells, including the decreased rate of cell and tumor growth (Figs. 3 and 5 and Supplemental Fig. S4). We determined expression levels of Ki67, a marker for cell proliferation, in tumor tissues originating from control and βArr2^ko^ SN12C cells. The sections from βArr2^ko^ tumors evidenced fewer Ki67-positive cells compared to control cells (Supplemental Fig. S5A), suggesting involvement of βArr2 in the cell cycle progression. LDHA, which is detected only in the human cells, was expressed similarly among control and βArr2^ko^ cells. To support the idea that βArr2 impacts the cell cycle, control and βArr2^ko^ cells were stained with DAPI, that reflects DNA content, and analyzed 48 hr after seeding. The majority of βArr2^ko^ cells were detected in sub-G1, outside the range of what was considered live for the control cells (Fig. 6A and Supplemental Fig. S5B). Of the live cells, we calculated the cell fraction in G1, S, and G2 phases and could observe the clear reduction in the number of βArr2^ko^ cells in G2 phase, in comparison to control cells (Fig. 6B).

Cyclin A is involved in the cell cycle progression and together with Cdk2 allows dividing cells to synthesize DNA and enter mitosis^27^. Indeed, Cyclin A expression is detectable in late G1 phase, continues to increase through S phase, reaches maximal levels in early G2 phase at which time the protein begins to degrade in the proteasome and becomes undetectable during early mitosis. Western blot analysis revealed that Cyclin A expression was reduced in βArr2^ko^ cells compared to the control cells (Fig. 6C). Distinctly, the levels of Cyclin D1, which is highly expressed in mid-G1 to early mitosis phases, were similar in the control and βArr2^ko^ cells. These observations are consistent with our results showing that the cell cycle arrest observed in βArr2^ko^ cells is at the S/G2 check point (Fig. 6B).

Progression through the cell cycle involves multiple intermediates, notable among them is the βArr effector tyrosine kinase c-Src^20,28,29^. We reasoned, therefore, that βArr2 regulates Cyclin A expression and cell cycle progression through c-Src activation. Indeed, we found that the knockout of βArr2 blunts the activation, but not expression, of c-Src as evidenced by the decreased c-Src phosphorylation on Y416 (Fig. 6D). Moreover, we observed that the treatment with PP2, a selective inhibitor of Src family kinases, decreased the expression of Cyclin A (Fig. 6E), implying βArr2 regulates the cell cycle progression through c-Src activation and Cyclin A expression.

## Discussion

Identification of molecular drivers of RCC is critical for the development of therapeutic strategies to more effectively treat the advanced disease. Here, we showed that βArr2 regulates the RCC cell proliferation *in vitro* and localized and metastatic tumor growth. Several studies have implicated βArr2 in mitogenic signaling^30^ and established its increased expression in human tumor versus normal tissue^31,32^. However, just how βArr2 expression and signal deregulation contributes to RCC remains unknown. Herein, we show that inhibition of βArr2 attenuates c-Src activation, Cyclin A expression, and the ensuing cell cycle progression that controls the cell growth (Fig. 6F).

The knockout of βArr2 inhibited the cell cycle progression and cell proliferation *in vitro*, and xenograft tumor formation in animals. In cell model systems, βArr2-mediated mitogenic signaling downstream of stimulated G protein-coupled receptors involves the activation of ERK MAP kinases and tyrosine kinases^10,33^. Consistent with these findings, our results here show that decreased expression of βArr2 inhibited c-Src activation. In the case of βArr1, it was reported that it binds directly to inactive c-Src leading to c-Src activation^20,28^. Our results show that downregulation of βArr2 inhibits c-Src activation but not expression in RCC cells, presumably due the lack of opportunity to form a βArr2-Src complex. In addition to the regulation of c-Src activation that is critical for cell proliferation, the knockout of βArr2 inhibited Cyclin A expression and cell cycle progression. The βArr2-dependent regulation of Cyclin A expression has not been reported before and the responsible mechanisms remain unclear. However, it has been reported that activated c-Src regulates cell cycle progression through Myc and Cyclin A/Cdk2, thereby permitting the S-G2 progression of the cell cycle^34^. Together, our results put forth an expanded role for βArr2 function in RCC cell growth; through the regulation of Cyclin A expression and cell cycle progression^35^.

RCC is a heterogeneous disease and the most associated genetic malfunction in ccRCC subtype is VHL inactivation^36^. However, VHL inactivation alone may not be sufficient to drive tumor formation as evidenced in genetically engineered Vhl^−/−^ animal models^37^. In addition to VHL inactivation, recent studies in mice have identified mutations in other genes, including cell cycle regulating genes, in the development of ccRCC^38^. Our study showed that knockout of βArr2 inhibits the cell cycle programs and RCC xenograft tumor growth in mice, suggesting deregulated expression of βArr2 may collaborate with disease-driving genetic mutations in the development of RCC. In a recent study, it was reported that ccRCC can develop in mice with deletion of Vhl, Trp53 and Rb1 genes^39^, but inactivation of Trp53 and Rb1 is rare in human ccRCC^3^. It is worth mentioning that tumor tissues from the Vhl/Trp53/Rb1 mouse expressed recurrent mutations in other genes, including Kif3a^39^, and βArr2 appears to regulate Kif3a function and cilium formation^40,41^. As such, our results are consistent with the idea that increased expression of βArr2 provides a supportive role in the development of ccRCC.

Ubiquitously expressed βArrestin proteins comprise βArr1 and βArr2 that are highly homologous and, for the most part, functionally redundant^33,42^. Our bioinformatics analyses revealed an association between increased expression of βArr2, and not βArr1, in RCC, implying functional specificity among the two related proteins. Indeed, proteomics analysis revealed specificity in the interaction between βArr1 and βArr2 and binding partners^43^. Using public databases we found that heightened expression of βArr2 positively correlates with RCC disease stage and poor prognosis thereby validating the clinical implication of βArr2 as a marker for advanced disease. Also, the increased expression of βArr2 in RCC patients is not restricted to race or gender and is detected in young patients^44^, reinforcing the idea that βArr2 may serve as an effective marker in the diagnosis of RCC.

In summary, we provide here clear evidence that βArr2 is highly expressed in human RCC tissue and exerts a key role in cancer formation. Inhibition of βArr2 expression reduced localized and metastatic RCC tumor growth. These results give rationale for the use of βArr2 as a prognostic biomarker and potential therapeutic target to combat the insofar lethal advanced RCC.

## Materials and Methods

### Reagents

Antibodies were obtained as follows: anti-βArr1/2 (D24H9), anti-βArr2 (C16D9), anti-human LDHA (3582), anti-phospho-Src (Y416) (D49G4), anti-Src (36D10), anti-Cyclin D1 (92G2), anti-GAPDH (2118S), and Signal Stain Boost IHC detection reagent from Cell Signaling; anti-human Ki67 (ab92742); anti-Actin (ab3280) from Abcam; anti-HSP90 (610419) from Fischer; anti-Cyclin A (H-432) from Santa Cruz Biotechnology; anti-Flag M2 (F3165) from Sigma; and HRP-coupled anti-rabbit (711-035-152) or anti-mouse (715-035-150) from Jackson Immuno Research Laboratories. Chemical and other reagents were obtained as follows: PP2 (Src family kinase inhibitor) from Selleckchem, protease inhibitor cocktail and puromycin from Sigma-Aldrich; polybrene from Millipore; collagen from Roche; matrigel from BD; Super Signal West Pico chemiluminescent substrate from Thermo Scientific; and Target Retrieval Solution, Protein Block, AEC substrate and Faramount aqueous mounting medium from Dako. High pure RNA isolation kit was from Roche, and iScript™ reverse transcription supermix for RT-qPCR and iQ SYBR green supermix were from Bio-Rad. Control and targeted siRNAs were from Dharmacon (SMARTpool: ON-TARGET plus ARRB2 siRNA) and shRNA bacterial glycerol stock clone ID: NM_004313.3-309s21c1 targeting ARRB2 was from Sigma.

### Mammalian cell culture

Human kidney cell lines HK2, CAKI-1, 786O, and ACHN were procured from the American Type Culture Collection and SN12C from the National Cancer Institute. All cells were maintained in RPMI 1640 medium (Corning) supplemented with 10% FBS (Sigma), 100 units/ml penicillin and 100 mg/ml streptomycin (Corning). The SN12C control (infected with lentiviral CRISPR/Cas9 vector containing no sgRNAs), ARRB2ex3-14, and ARRB2ex4-12.19 (infected with CRISPR/Cas9 lentiviral vector with sgRNA targeting exon 3 [(+) strand: 5′-GCG GGA CTT CGT AGA TCA CC-3′-TGG (PAM)] or exon 4 [(+) strand: 5′-GAC TAC CTG AAG GAC CGC AA-3′-AGG (PAM)] of ARRB2 gene respectively) were engineered and maintained with 1 μg/ml puromycin.

### RNA extraction and expression

Total RNA was extracted with High Pure RNA isolation kit (Roche), and 1 μg in a final volume of 20 μl was reverse-transcribed with iScript™ Reverse Transcription Supermix per the manufacturer’s instructions. Quantitative PCR reactions containing 400 ng of cDNA and 5 μl of iQ SYBR Green Supermix 5× in a total volume of 10 μl were performed in triplicate using Bio-Rad CFX detection system and target gene expression was normalized to 18S RNA. The primers used for gene amplification of human 18S (QT00199367), ARRB1 (QT00071197) and ARRB2 (QT00058051) were obtained from Qiagen.

### Immunoblotting

Cells were lysed with RIPA buffer (50 mM Tris-HCl pH 7.4, 150 mM NaCl, 2 mM EDTA, 1% NP-40, and 0.1% SDS) and fresh protease inhibitor cocktail. Total cell lysates (25 μg/lane) were separated on 8% SDS-PAGE and electrophoretically transferred to nitrocellulose membranes. The membranes were blocked in 4% BSA in PBST at room temperature for 1 hr, incubated with primary antibodies (1:1000) at 4 °C for 16 hr, followed by incubation with HRP-coupled secondary antibodies (1:30,000) for 1 hr. Specific bands were visualized with SuperSignal West Pico chemiluminescent substrate, and blots were imaged with Kinoca Minilia SRX-101A processor. Band intensities were measured using ImageJ software.

### Migration and invasion assays

Cell migration assays were done using 8 μm pore transwell chambers (Fischer; 07-200-150). Briefly, cells were serum starved for 16 hr, detached, re-suspended in starvation medium, and added to the transwell chambers (2.5 × 10^4^ cells/well for ACHN, and 3 × 10^4^ cells/well for SN12C). Starvation medium containing 1% FBS was added to the lower chambers and incubated at 37 °C for 8 hr for ACHN and 24 hr for SN12C cells. For invasion assay, cells (1 × 10^5^ cells/well for ACHN, and 5 × 10^4^ cells/well for SN12C) were seeded on pre-coated matrigel inserts (BD Biosciences; 08-774-122) and incubated for 24 hr. At termination point, cells were fixed and stained with 0.1% crystal violet in 20% ethanol. Cells that remained at the top of filter were removed and migrated cells were counted through a 10× objective lens with an Axioskop microscope (Zeiss).

### Tumor implantation

All experiments involving mice were done according to a protocol reviewed and approved by the Institutional Animal Care and Use Committee at the University of Florida. Subrenal capsule implantation protocol is detailed in Zhang *et al*.^26^. Briefly, male hsd: athymic nude-Fox^1nu^ (Envigo), 6 weeks old mice were grouped according to body weight. Soft collagen pellets containing 1 × 10^6^ cells were placed under the capsules of the left kidney. Tumor growth was monitored by palpation and ultrasound imaging (GE Medical System InSiteExC) and, at the end of the experiment, both kidneys, draining lymph nodes, spleen, liver, intestine, and lungs were harvested, weighed, and fixed in 10% buffered formalin phosphate for IHC analysis.

### Immunohistochemistry

Harvested organs were embedded in paraffin and sectioned (5 μm), deparaffinized in xylene, rehydrated in graded alcohol, subjected to heat-induced antigen retrieval, and blocked. Sections were probed with the indicated antibody at 4 °C overnight, and then incubated with Signal Stain Boost. Samples were developed with AEC substrate, counterstained with hematoxylin, and mounted with Faramount. Microscopic images were taken at 10× or 40× using Nikon Eclipse 50i microscope equipped with a DS-Fi1 camera and NIS-Elements BR3.1 software.

### Statistics

Data are expressed as means ± SEM or SD. Statistical analysis was performed with either one-way ANOVA with Tukey’s post-test or two-tailed paired Student’s t test, and a P < 0.05 was considered statistically significant. Graphs were generated using Graph Pad Prism 6 software and axis labels were generated using Adobe Illustrator CS5.1.

## Electronic supplementary material

Supplementary Information

Supplementary Information
