# Supplementary material for: βArrestin2 Mediates Renal Cell Carcinoma Tumor Growth
Source: Sci Rep. 2018 Mar 20;8:4879. doi: 10.1038/s41598-018-23212-w (PMC5861056; doi:10.1038/s41598-018-23212-w)
Supplement: Supplementary file 1 — Supplementary Information [file 41598_2018_23212_MOESM1_ESM.pdf]

Supplemental Information

## βArrestin2 Mediates Renal Cell Carcinoma Tumor Growth

Jude Masannat[‡], Hamsa Thayele Purayil, Yushan Zhang[†], Michelle Russin, Iqbal Mahmud, Wanju Kim, Daiqing Liao, Yehia Daaka[§]

Department of Anatomy and Cell Biology, University of Florida College of Medicine, Gainesville, FL 32610

[§]Correspondence: Yehia Daaka, 1333 Center Drive, B1-004, Gainesville, FL, 32610. Phone: 352-273-8112; Fax: 352-846-1248; E-mail: ydaaka@ufl.edu

**Supplementary Figure Legends**

**Figure S1. ARRB2 expression profile in human RCC.** ARRB2 transcript levels from four Gene Expression Omnibus (GEO) datasets of **A,** GSE11151; **B**, GSE15641; **C,** GSE14994; and **D,** GSE6344 were queried for the association with disease status. P values were calculated by using two-tailed Student's t test. Box and whisker plot line (from top to bottom): maximum; Q3, third quartile; median; Q1, first quartile; and minimum.

**Figure S2. βArr2 mediates RCC cell migration and invasion. A,** Forced expression of βArr2 in CAKI-1 cells. Cells were transfected with empty vector pcDNA (EV) or pcDNA-Flag-βArr2 (Flag-βArr2) plasmids. Western blots show expression of βArr2. HSP90 expression served as a protein loading control. **B,** Knockdown of βArr2 in ACHN cells. Cells were transfected with siβArr2 (50 nM) and control (siCon) and βArr2 protein levels were measured by Western blot. **C**, Migration assay. Migration assay using control and βArr2 knockdown ACHN cells were performed for 8 hr using transwell inserts. Images shown are representative of three independent trials. **D**, Migrated cells in five randomly-selected 100x fields were counted and plotted relative to control cells. **E,** Invasion assay. Control and βArr2 knockdown SN12C ($1.0 \times 10^5$) cells were allowed to migrate through matrigel-coated transwell inserts for 24 hr and then invaded cells were quantified. Data shown are mean ± SD for three trials, $^{**}P < 0.001$, $^{***}P < 0.0001$.

**Figure S3. RCC localized and metastatic tumor growth are controlled by βArr2. A,** Representative ultrasound images of mice kidneys after tumor implantation. Tumor growth images are from the same mouse tracked over time. Orange circle shows the tumor and the circle size reflects potential tumor area. An image of the normal contralateral kidney is also shown for the same mouse. **B**, A representation of the overall visible metastasis status at experiment termination for control and $\beta Arr2^{ko}$ cells implanted mice. Arrows indicate metastatic growth. **C**, Renal lymph nodes from control and $\beta Arr2^{ko}$ Clone1 and Clone2 were harvested

from mice (n=7 mice per cell line) and pictured. N, contralateral normal kidney and T, kidney harboring tumor. **D**, Tumor weight was measured by subtracting the weight of N lymph node from that of T lymph node. **, $P < 0.01$. **E**, Representative anti-human LDHA staining of lymph node tissue sections showing the infiltration to tumor-side and contralateral-side lymph node of control, but not $\beta Arr2^{ko}$ Clone1 or Clone2 harboring animals.

**Figure S4. RCC tumor growth is controlled by βArr2. A,** Subrenal tumor growth. Control and $\beta Arr2^{ko}$ cells were embedded in collagen and implanted under the renal capsule of Hsd: athymic nude mice and allowed to grow for 9 weeks (N-contralateral normal kidney, T-kidney with tumor). **B,** Scatter-plot showing the tumor weights of 5 week-long and 9 week-long subrenal tumor growth calculated by subtracting the weight of normal kidney (N) from tumor kidney (T).

**Figure S5. βArr2 regulates cell cycle progression. A**, Proliferation assay. Representative IHC images of anti-human Ki67 staining and consecutive anti-human LDHA staining of the same tumor area are shown. **B,** Cell cycle analysis. Control and $\beta Arr2^{ko}$ ($5 \times 10^4$) cells were counted in the LSRII flow machine and plotted against FSC. Blue bar represents gating for viable cells and the percentage of cells counted in the gate is indicated above the bar.

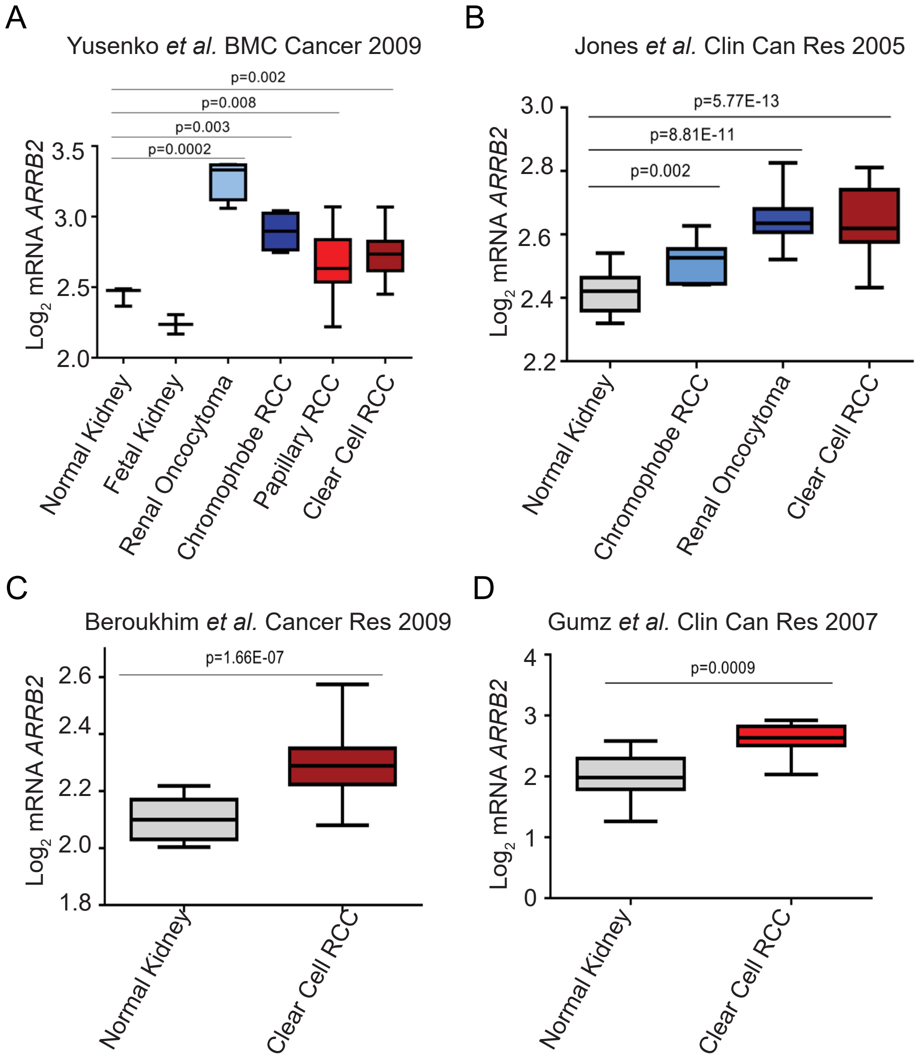

A
Yusenko et al. BMC Cancer 2009
p=0.002
p=0.008
p=0.003
p=0.0002
Log2 mRNA ARRB2
3.5
3.0
2.5
2.0
Normal Kidney
Fetal Kidney
Renal Oncocytoma
Chromophobe RCC
Papillary RCC
Clear Cell RCC
B
Jones et al. Clin Can Res 2005
p=5.77E-13
p=8.81E-11
p=0.002
Log2 mRNA ARRB2
3.0
2.8
2.6
2.4
2.2
Normal Kidney
Chromophobe RCC
Renal Oncocytoma
Clear Cell RCC
C
Beroukhim et al. Cancer Res 2009
p=1.66E-07
Log2 mRNA ARRB2
2.6
2.4
2.2
2.0
1.8
Normal Kidney
Clear Cell RCC
D
Gumz et al. Clin Can Res 2007
p=0.0009
Log2 mRNA ARRB2
4
3
2
1
0
Normal Kidney
Clear Cell RCC

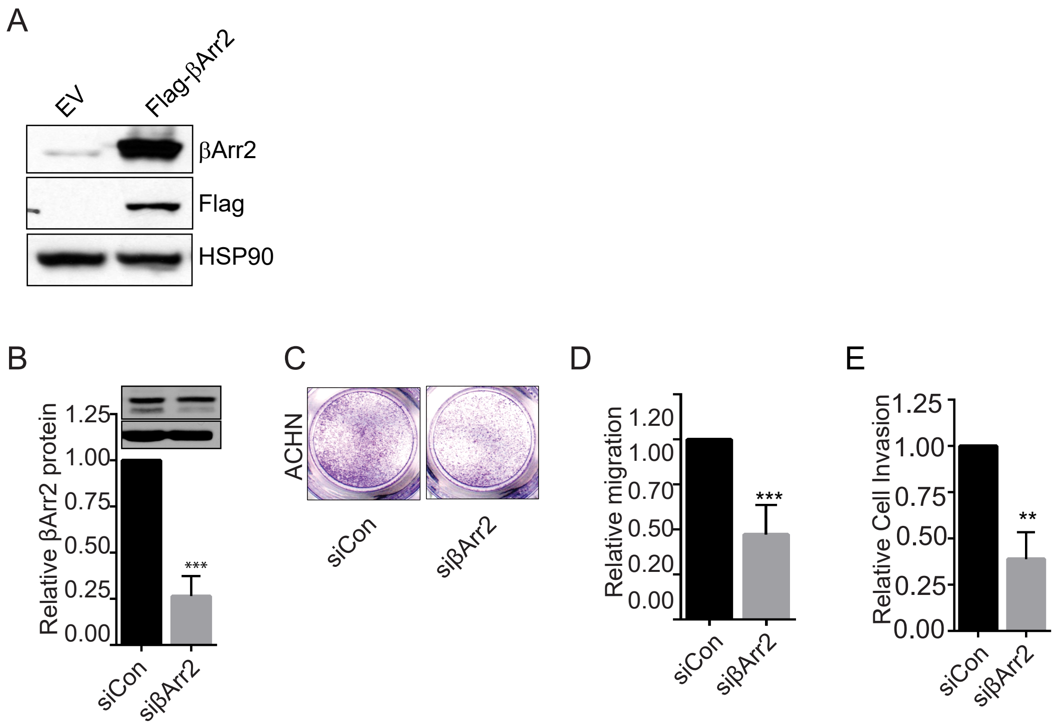
A
EV
Flag-βArr2
βArr2
Flag
HSP90
B
Relative βArr2 protein
1.25
1.00
0.75
0.50
0.25
0.00
***
siCon
siβArr2
C
ACHN
siCon
siβArr2
D
Relative migration
1.20
1.00
0.70
0.50
0.20
0.00
***
siCon
siβArr2
E
Relative Cell Invasion
1.25
1.00
0.75
0.50
0.25
0.00
**
siCon
siβArr2

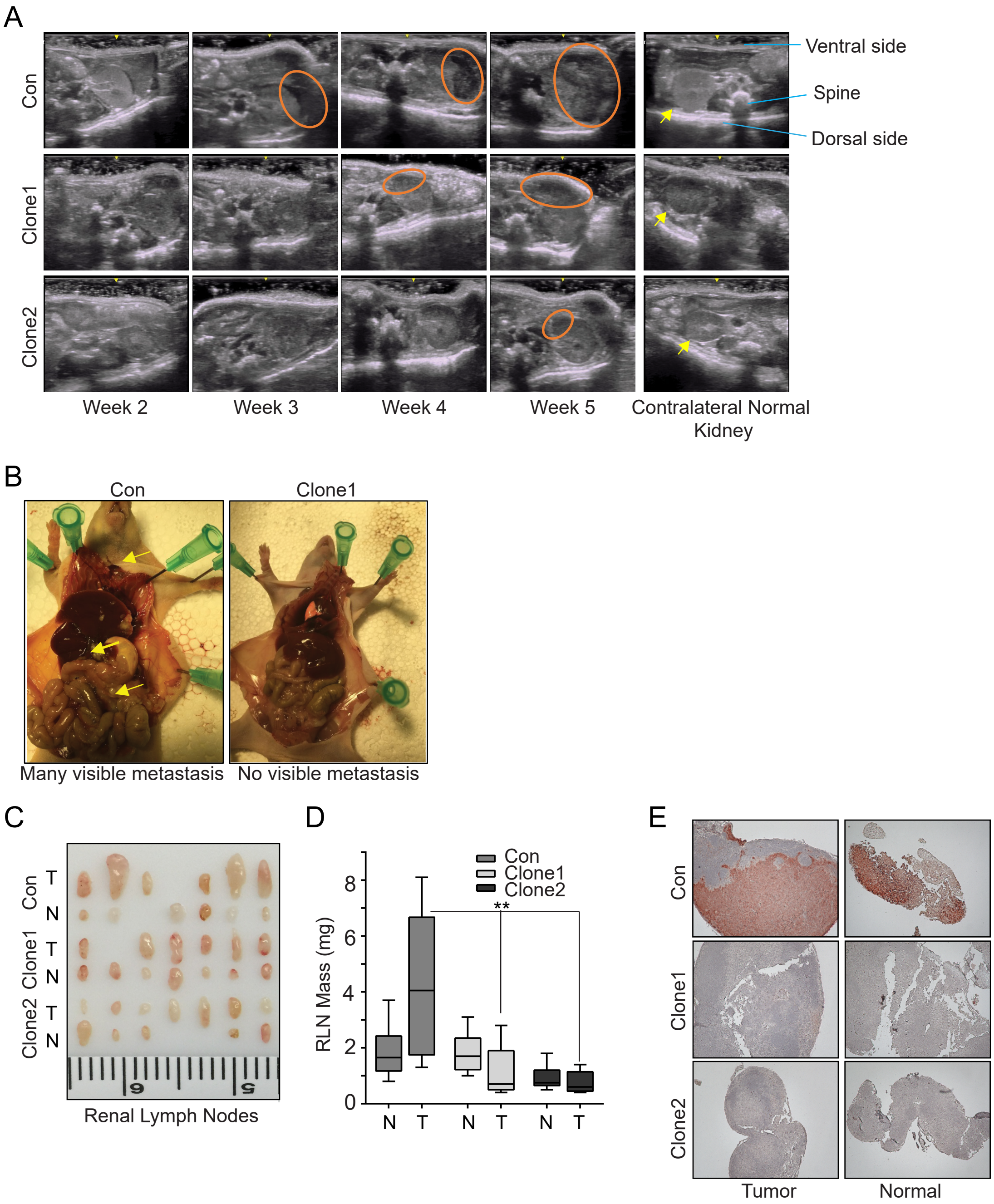

A
Con
Clone1
Clone2
Ventral side
Spine
Dorsal side
Week 2
Week 3
Week 4
Week 5
Contralateral Normal Kidney
B
Con
Clone1
Many visible metastasis
No visible metastasis
C
Con T N
Clone1 T N
Clone2 T N
Renal Lymph Nodes
D
Con
Clone1
Clone2
**
RLN Mass (mg)
0
2
4
6
8
N T N T N T
E
Con
Clone1
Clone2
Tumor
Normal

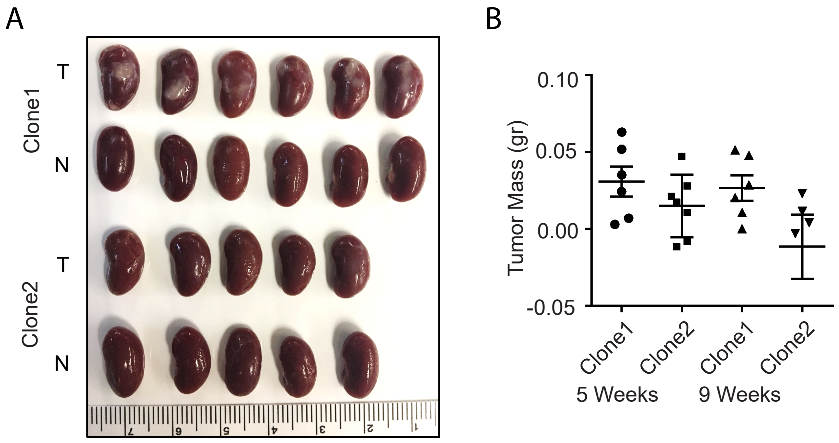

A
B
Clone1
T
N
Clone2
T
N
Tumor Mass (gr)
0.10
0.05
0.00
-0.05
Clone1
Clone2
Clone1
Clone2
5 Weeks
9 Weeks

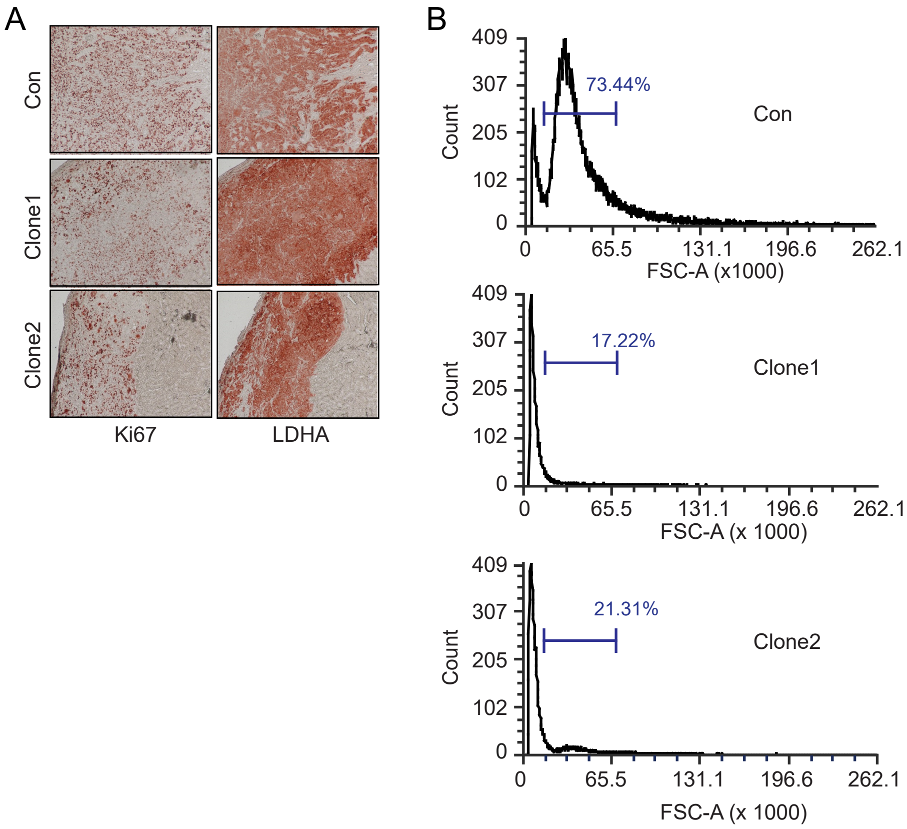
A
Con
Clone1
Clone2
Ki67
LDHA
B
409
307
205
102
0
Count
73.44%
Con
0
65.5
131.1
196.6
262.1
FSC-A (x1000)
17.22%
Clone1
FSC-A (x 1000)
21.31%
Clone2
FSC-A (x 1000)
