# Supplementary material for: βArrestin2 Mediates Renal Cell Carcinoma Tumor Growth
Source: Sci Rep. 2018 Mar 20;8:4879. doi: 10.1038/s41598-018-23212-w (PMC5861056; doi:10.1038/s41598-018-23212-w)

Supplemental Information: Raw Data

βArrestin2 Mediates Renal Cell Carcinoma Tumor Growth

Jude Masannat[‡], Hamsa Thayele Purayil, Yushan Zhang[†], Michelle Russin, Iqbal Mahmud, Wanju Kim, Daiqing Liao, Yehia Daaka[§]

Department of Anatomy and Cell Biology, University of Florida College of Medicine, Gainesville, FL 32610

[§]Correspondence: Yehia Daaka, 1333 Center Drive, B1-004, Gainesville, FL, 32610. Phone: 352-273-8112; Fax: 352-846-1248; E-mail: ydaaka@ufl.edu

Figure 3A

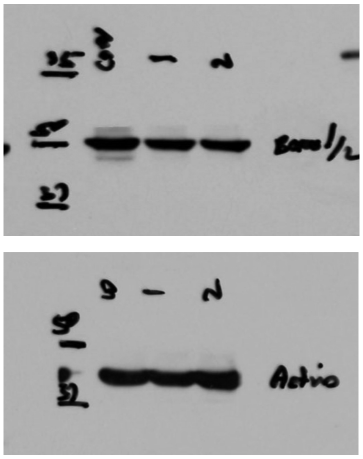

Figure 3C

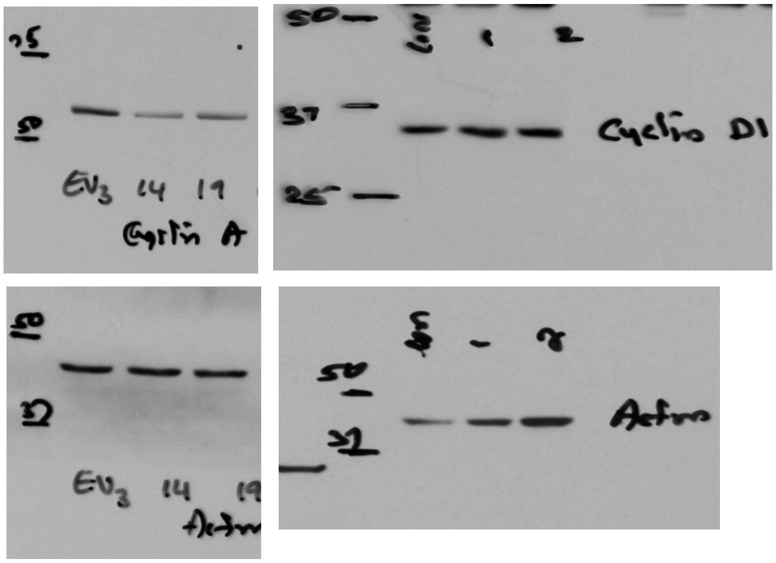

Figure 6E

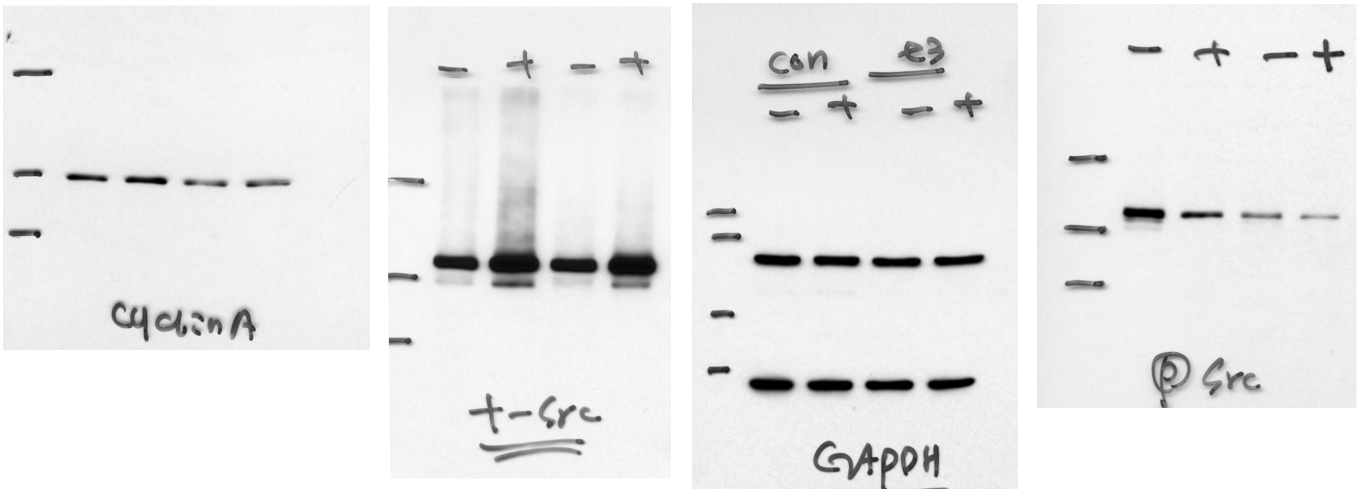

Supplement: Supplementary file 2 — Supplementary Information [file 41598_2018_23212_MOESM2_ESM.pdf]
